# Movement Disorder Change in a Child with Choreic Movement Disorder Using an Elasto-Compressive Bodysuit

**DOI:** 10.3390/children13050600

**Published:** 2026-04-27

**Authors:** Domenico M. Romeo, Chiara Velli, Francesca Sini, Maddalena Bianchetti, Eugenio Mercuri

**Affiliations:** 1 Pediatric Neurology Unit, Fondazione Policlinico Universitario A. Gemelli IRCCS, 00168 Rome, Italy; 2 Pediatric Neurology Unit, Università Cattolica del Sacro Cuore Roma, 00168 Rome, Italy

**Keywords:** cerebral palsy, movement disorders, dynamic movement orthoses, dyskinetic, rehabilitation

## Abstract

**Background/Objectives**: Dynamic movement orthoses (DMOs) are elasto-compressive bodysuits used in the rehabilitation of children with motor disabilities, mainly in children with cerebral palsy. Among the DMOs, the FLEXA^®^ represents one of the most frequently used orthoses in clinical practice due to its adaptability and flexibility. The purpose of this case study is to describe the application of FLEXA^®^ in an 18-month-old female child with choreic cerebral palsy. **Methods**: To evaluate the effect of the dynamic movement orthosis (FLEXA^®^), the *Movement Disorder-Childhood Rating Scale* (MD-CRS) 0–3 years was administered. The child was evaluated before the use of the FLEXA^®^ bodysuit, and with the bodysuit donned approximately 30 min after its application. A follow-up using the same assessments was carried out at 6 months. **Results**: The results showed an important change in the severity of the movements according to the MD-CRS; mainly, the child’s movement disorder severity changed from a grade five severity (profoundly affected) without the bodysuit to grade three (moderately affected) with the use of the bodysuit. The benefit was maintained during follow-up even without the bodysuit, and a further improvement was observed in assessments with the bodysuit. **Conclusions**: This case report highlights the potential benefits of dynamic movement orthosis, like the FLEXA^®^, in managing movement disorders in children with choreic form of cerebral palsy. Extending the study to a larger sample would help to strengthen the validity of these findings and confirm the beneficial effects of use of DMOs for children with movement disorders.

## 1. Introduction

Cerebral palsy (CP) is one of the most common physical disorders of childhood, defined as a group of permanent disorders of movement and posture development, causing activity limitation, that are attributed to non-progressive disturbances that occur in the developing fetal or child brain [1,2].

Depending on the type of movement disorder, cerebral palsy is classified as spastic, dyskinetic, including dystonia and athetosis, ataxic, or hypotonic. The dyskinetic forms represent about 10% of cerebral palsy cases; these are characterized by abnormal movements, abrupt changes in tone, and the persistence of primitive reflexes that limit the ability to organize and execute voluntary movements and the ability to maintain posture. The dyskinetic form of CP represents one of the most frequent forms of movement disorder in childhood [3] and it is frequently associated with disorders of the cerebral cortex, basal ganglia, cerebellum and other motor pathways, which are the cause of a brain injury [4]. Dyskinetic CP is also typically associated with choreic movements, characterized by involuntary, rapid, jerky, and unpredictable motions; these movements are often fidgety, chaotic, or dance-like, affecting the limbs and face while worsening with stress [3,4].

In alignment with the WHO International Classification of Functioning, Disability and Health (ICF), the primary goal of managing children with dyskinetic cerebral palsy is to enhance daily functioning and quality of life by optimizing motor control and posture, while mitigating associated impairments, pain, and discomfort [5,6].

According to the recent literature, the most evident therapeutic approaches for the rehabilitation of dyskinetic forms of cerebral palsy are taping, mobility, strength training and environmental enrichment to promote task performance [7,8,9].

However, as widely supported by the literature, the cornerstone of managing children with dyskinetic cerebral palsy is a multidisciplinary approach. The synergy between physiotherapists, occupational therapists and speech therapists facilitates the development of a personalized rehabilitation program, ensuring early intervention [5].

Pharmacological treatments, using baclofen, tetrabenazine, trihexyphenidyl, botulin toxin and benzodiazepines, are also proposed, even in young patients with dyskinetic CP [10,11,12,13].

Within such a rich and complex framework, dynamic movement orthoses (DMOs) are elasto-compressive tools used in the rehabilitation of children with motor disabilities and in children with cerebral palsy [8]. The literature review on preventive and rehabilitative interventions in children with cerebral palsy identifies the use of DMOs as an intervention that is likely to benefit their motor function [7]. The aim of these orthoses is to improve postural stability, posture symmetry, and movement, without restricting functional movement. The elastic fabric consists of individual panels sewn together to apply pressure to the trunk and limbs, combined with zips to allow for ease of application. The use of DMOs to support the rehabilitation of children with motor disabilities has been extensively used in clinical practice and in various scientific works to promote normalization of muscle tone, increased perception of body position in space, symmetry, posture and movement, stability and proximal control, and the reduction of pain [14,15].

Many types of DMOs are used in clinical practice either in conjunction with standard therapy or within specific treatment programs. These include the Theratogs suit, Adeli suit treatment, Lycra, FLEXA^®^, SPIO, Spinecor, and SelLi suit [8]. These suits are dynamic orthoses available in different designs from full bodysuits to smaller garments such as sleeves/gloves and leggings. Although there is no consensus regarding the advantages and disadvantages of wearing these suits, they are used in clinical practice as part of a specific rehabilitation program and monitored by therapists [8].

### FLEXA^®^

Among the DMOs, the FLEXA^®^, manufactured in Italy, is one of the most frequently used in our clinical practice due to its adaptability and flexibility; it can be applied as a bodysuit, glove, or tight-fitting legging that completely covers the trunk, arms, and legs. FLEXA^®^ is less invasive than traditional orthoses, is breathable, and has a wide weave. It has seven lines of force and can be reinforced to exert specific directional forces without restricting functional movement. The pressure applied by the body improved stability and decreased the effect of external force vectors. It can contain and reduce involuntary movements, favoring realignment and giving the muscles a biomechanical advantage. This type of DMO is distinguished from others by the use of innovative materials and attention to packaging, elements that contribute to comfort and resistance. The base of the bodysuit is made of a natural-synthetic fiber. Cotton is the component in direct contact with the skin. A significant aspect of the FLEXA^®^ is the use of elasticized velcro, which allows prolonged brace use, and adapts to the patient’s growing and changing needs. The materials used for the FLEXA^®^ are supplied by European manufacturers (Germany and Italy).

A further advantage offered by the FLEXA^®^ is the possibility of customization, responding to the requests of physicians and therapists and providing a personalized solution for each patient.

Despite its large use in clinical practice, the specific use of FLEXA is reported exclusively in a case report that describes the usage of a FLEXA glove in a boy with cerebral palsy as an alternative to neuromuscular taping to increase autonomy and independence [16].

The purpose of the present case report is to describe the application of FLEXA^®^ in a female child of 18 months of correct age with a choreic form of cerebral palsy to improve her movement disorder.

## 2. Materials and Methods

This case report details on the application of FLEXA^®^ in a female child, corrected age (CA) 18 months, with cerebral palsy, specifically with a choreic movement disorder. The child was born prematurely at 32 weeks and 6 days gestation due to premature rupture of membranes and was delivered urgently via cesarian section. At term age, an MRI indicated extensive gliotic–malacic changes, cortical necrosis, and hemosiderin deposits primarily in the posterior periventricular white matter and basal nuclei. By 4 months CA, a neurological structured evaluation [2] identified motor delays with a predominant extensor tone at the axial level and a significant visual deficit. The child used Valproic acid from 9 months CA due to the presence of electroclinical episodes characterized by focal onset epileptic spasms. During follow-up, psychomotor development was assessed using the Bayley-III scale at 12 months CA, which revealed delays in cognitive and language development, as well as in socio-emotional behavior and adaptive functioning. Genetic analyses for isolated congenital heart defects and array comparative genomic hybridization (array-CGH) were conducted, with normal results. At 12 months CA, the child exhibited significant axial hypotonia, poor trunk control, and chaotic, uncoordinated movements.

At 15 months CA, the child shows a level V in the Gross Motor Function Classification System E & R (12% at Gross Motor Function System-66) [1,17,18] and the motility evolved into a choreiform movement disorder affecting the trunk, limbs, and peribuccal area, both at rest and during task performance; symptom fluctuations were also reported by parents, influenced by stress, fatigue, and were ceasing during deep sleep. The child was involved in a neuromotor rehabilitation 3 times/week from the age of 2 months. Due to the movement disorder impacting motor performance, the family was introduced to the use of a DMO (FLEXA^®^) from 18 months CA, to improve axial control, alignment, and stabilization. An occupational therapist performed the measures and fitted the FLEXA^®^; this suit features seven specific lines of force to apply directional pressure to the muscles of the trunk. The therapist also educated the family on its use for at least 4–6 h/day during everyday routine activities. At the time of the introduction of the DMO, the child was not taking medication for chorea.

### Outcomes

The subsequent follow-up noted good compliance with the bodysuit, improved postural stability and reduced abrupt limb movements. To evaluate the effect of the DMO (FLEXA^®^), the movement disorder-Childhood Rating Scale (MD-CRS) 0–3 scale was used [19]. This is an age-specific scale for infants from 0 to 3 years which recognizes the presence of the movement disorder by pointing out its prevalent pattern; it also evaluates how much the movement disorder affects motor functions and activities of daily living, and rates the intensity of the disorder in different body regions. The MD-CRS allows monitoring of the disorder over time and the effectiveness of pharmacological, surgical or rehabilitative treatment [19]. The scale is composed of two sections: general assessment (Part I) and movement disorder (MD) assessment (Part II). Part I’s purpose is to allow a global assessment of the child from observing how much the movement disorder affects motor function, oral/verbal function, level of responsiveness to the environment, and in older children, personal autonomies. Part II assesses the severity of the movement disorder in different parts of the body. Scoring the scale to generate an Index I related to Part I (general assessment), an Index II related to Part II (movement disorder severity), and a Global Index. Each Index can range from 0 to 1, and it is divided into five classes according to the severity of the movement disorder. Class 1 included a 0–0.2 Index (healthy), class 2 a 0.2–0.4 Index (mildly affected), class 3 a 0.4–0.6 Index (moderately affected), class 4 a 0.6–0.8 Index (severely affected), and class 5 a 0.8–1 Index (profoundly affected). The MD-CRS scale is validated and extensively used in clinical practice for the evaluation of rehabilitative and pharmacological treatments due to its easy reproducibility and its inter- and intra-rater reliability [19,20]. The Movement Disorder-Childhood Rating Scale (MD-CRS) 0–3 was administered to the child twice (T0 and T1) on the same day: once without the bodysuit (T0) and then with the bodysuit on after wearing it for approximately 30 min after its application in which the infant performed normal playing activities (T1). The same assessment was performed at the 6-month follow-up, in order to assess the persistence of the observed benefits and evaluate any further improvements in the clinical condition. In the follow-up assessment, the evaluation was also conducted with and without the bodysuit (T2 and T3). During the follow-up period, the child continued to use the body for at least 4–6 h/day during everyday routine activities. The assessment was administered by one pediatric therapist (CV) trained in the use of the Movement Disorder-Childhood Rating Scale (MD-CRS). Written parental consent for publication was obtained.

## 3. Results

Administration of the MD-CRS 0–3 with and without the bodysuit, (T0–T1), showed a significant change in the severity of the movement disorder (Index II) (Table 1, Supplementary Materials Video S1). The severity of the child’s movement disorder (Part II) with the use of the bodysuit was placed in class three (classified as “moderately affected by MD”) in comparison with the assessment without the use of the bodysuit, which showed a class five for severity (classified as “profoundly affected by MD”) (Table 1). In detail, the improvements were observed in the trunk, upper limb, and lower limb items. However, no differences were found in Part I (general assessment) and Global Index with and without the bodysuit.

Administration of the MD-CRS 0–3, at the follow-up visit, with and without the bodysuit (T2–T3), showed a significant change in the severity of the movement disorder (Index II) and in Global Index (Table 2). The severity of the child’s movement disorder (Part II) with the use of the bodysuit was placed in class two (classified as “moderately affected by MD”) in comparison with the assessment without the use of the bodysuit, which showed a class three for severity (classified as “moderately affected by MD”) (Table 2). The Global class of the scale with the use of the bodysuit was placed in a class three (classified as “moderately affected by MD”) in comparison with the assessment without the use of bodysuit, which showed a class four (classified as “severely affected” by MD). As reported at T0 and T1 assessments, no differences were observed in Part I (general assessment) between participants wearing the suit and those not wearing it.

A change in the severity of the child’s movement disorder (Part II) was observed between the baseline assessment without the bodysuit (T0) and the follow-up assessment (T2), and in comparison with the assessments conducted with the bodysuit (T1–T3). Moreover the Global class showed a change in the evaluation at baseline and in the follow-up with the bodysuit (T1–T3). At T3, the Global class of the scale was placed in a class three (classified as “moderately affected” by MD) in comparison with the assessment at T1, which showed a class four (classified as “severely affected” by MD).

The child tolerated the bodysuit well, with no reported adverse effects. There were no reports of difficulties in managing toileting by the parent.

## 4. Discussion

This case report is the first study presenting the use of a FLEXA^®^ orthosis in a pediatric choreiform movement disorder.

These patients experience involuntary, fluctuating muscle tone (stiffness to floppiness) and random movements, often causing severe and uncontrollable writhing or jerky motions of the limbs and face. This disruption profoundly affects proprioception and sensory processing due to the altered sensorimotor cortical activation and disrupted sensory pathways and leading to significant challenges with balance, posture, and motor control [3,4].

In the present case study, FLEXA^®^, a specific type of DMO, was used to improve the movement disorder. In particular, an 18-month-old female child with a choreic movement disorder showed a significant improvement in clinical assessment with the MD-CRS after wearing a FLEXA ^®^ bodysuit for 30 min, compared to the evaluation without the bodysuit. The greatest clinical change was observed in the assessment of the severity of the presence of the movement disorder (Index II) with a change of two classes, especially in the trunk, upper limbs, and lower limbs. At the 6-month follow-up, these changes persisted even without the bodysuit and showed further improvement with bodysuit with a change in Global class. Moreover, at follow-up, the use of the bodysuit was associated with an increase in the global score of the MD-CRS.

Our results showed that wearing the FLEXA^®^ produced some immediate improvement of movement disorder, with a further improvement at 6 months not only in the assessments performed with the child wearing the bodysuit but also in the assessment when the child was not wearing the suit. This suggests that the FLEXA^®^ may produce an overall effect that is maintained over months, placing it as a potential intervention rather than a possible effect of maturation or ongoing physiotherapy at the 6-month follow-up. This is proved by a further improvement of the movement disorder when the child wore the bodysuit at follow-up (T3).

The MD-CRS is considered a sensitive tool to detect changes and as a possible outcome measure during natural history data collection or during clinical trials in children with movement disorders [12,19,20,21,22,23]. The Minimal Detectable Difference (MDD) values were also previously calculated with values between 0.05 and 0.17 (25). The observed change in Index II in the present case study exceeds the MDC and therefore appears to be of clinical significance.

The efficacy of FLEXA^®^ for movement disorders, represented by the reduction in movement in the examined patient, may be related to the proprioceptive stimulus given by the orthosis. The FLEXA^®^ acts like a second skin, providing a feeling of containment and a perceptive margin without restricting movement. Pressure from skin contact and increased internal soft tissue pressure leads to enhanced proprioceptive feedback to improve positional limb and body awareness, improving muscle activation and movement control. This dynamic orthosis also provides a proprioceptive stimulation that supports movement especially in children with dyskinetic disorders [21,22,23].

An intensive therapy with the suit on can enable re-education of the brain to recognize and form the correct movement of the muscles. The increase in correct proprioceptive input results in an increase in proper alignment [23].

### Limitation

As a case report, the present study presents several limitations with low generalizability to broader populations. The assessments (T0 and T1) were performed on the same day, by the same therapist, who was aware of the study objective. The 30 min effect may reflect a mechanical effect and/or a sensory/proprioceptive effect, although the follow-up assessments seem to confirm the role of FLEXA^®^ in improving the movement disorder. Furthermore, no objective kinematic measures were performed.

However, the present case study could help to develop new hypotheses on rehabilitation and everyday life settings that can later be tested quantitatively.

In this scenario, the results on the use of FLEXA ^®^ on this child seem encouraging, even though a follow-up is mandatory to confirm the beneficial effects of continuous use of the DMO over both short and long periods of time. It is also necessary to increase knowledge about the use of these orthoses in children with movement disorders by expanding the study sample.

## 5. Conclusions

In conclusion, this case report highlights the potential benefits of dynamic movement orthosis like FLEXA^®^ in managing movement disorders in children with the choreic form of cerebral palsy. Given an observed reduction in movement disorder affecting the four limbs and trunk in a timeframe of 30 min, and the persistence of this reduction also without the bodysuit at the follow-up, the authors proposed the prolonged use of this DMO to analyze the persistence of these achievements to promote rehabilitative goals. A reduction in limb and trunk movement disorders may transiently reduce movement severity and facilitate the development of gross motor skills. The possibility of a positive impact on rehabilitative goals is also supported by the good effects with and without the bodysuit at the follow-up. However, functional implications remain to be determined.

## Figures and Tables

**Table 1:** Movement Disorder Child Rating Scale results.

	Index I	Class I	Index II	Class II	Global Index	Global Class
Without Bodysuit (T0)	0.675	4 (Severely affected)	0.857143	5 (Profoundly affected)	0.75	4 (Severely affected)
With Bodysuit (T1)	0.675	4 (Severely affected)	0.571429	3 (Moderately Affected)	0.632353	4 (Severely affected)

**Table 2:** Movement Disorder Child Rating Scale results—Follow-up.

	Index I	Class I	Index II	Class II	Global Index	Global Class
Without Bodysuit (T2)	0.7	4 (Severely affected)	0.05	3 (Moderately affected)	0.61765	4 (Severely affected)
With Bodysuit (T3)	0.65	4 (Severely affected)	0.28571	2 (Mildly Affected)	0.5	3 (Moderately Affected)

## Data Availability

The original contributions presented in this study are included in the article/Supplementary Materials. Further inquiries can be directed to the corresponding author.

## Supplementary Materials

The following supporting information can be downloaded at https://www.mdpi.com/article/10.3390/children13050600/s1. Video S1. Video of MD-CRS with/without Flexa.

## Abbreviations

The following abbreviations are used in this manuscript:

DMO	Dynamic Movement Orthoses
MD-CRS	Movement Disorder-Childhood Rating Scale
CP	Cerebral Palsy
CA	Correct Age
Array-CGH	Array Comparative Genomic Hybridization
MD	Movement Disorder
GMFM	Gross Motor Function Measure
PEDI	Pediatric Evaluation of Dysability Inventary
QUEST	Quality of Upper Limb Skills Test
VAS	Visual Analog Scale
